# Supplementary figures and images for: The cJUN NH2-terminal kinase (JNK) signaling pathway promotes genome stability and prevents tumor initiation
Source: eLife. 2018 Jun 1;7:e36389. doi: 10.7554/eLife.36389 (PMC5984035; doi:10.7554/eLife.36389)

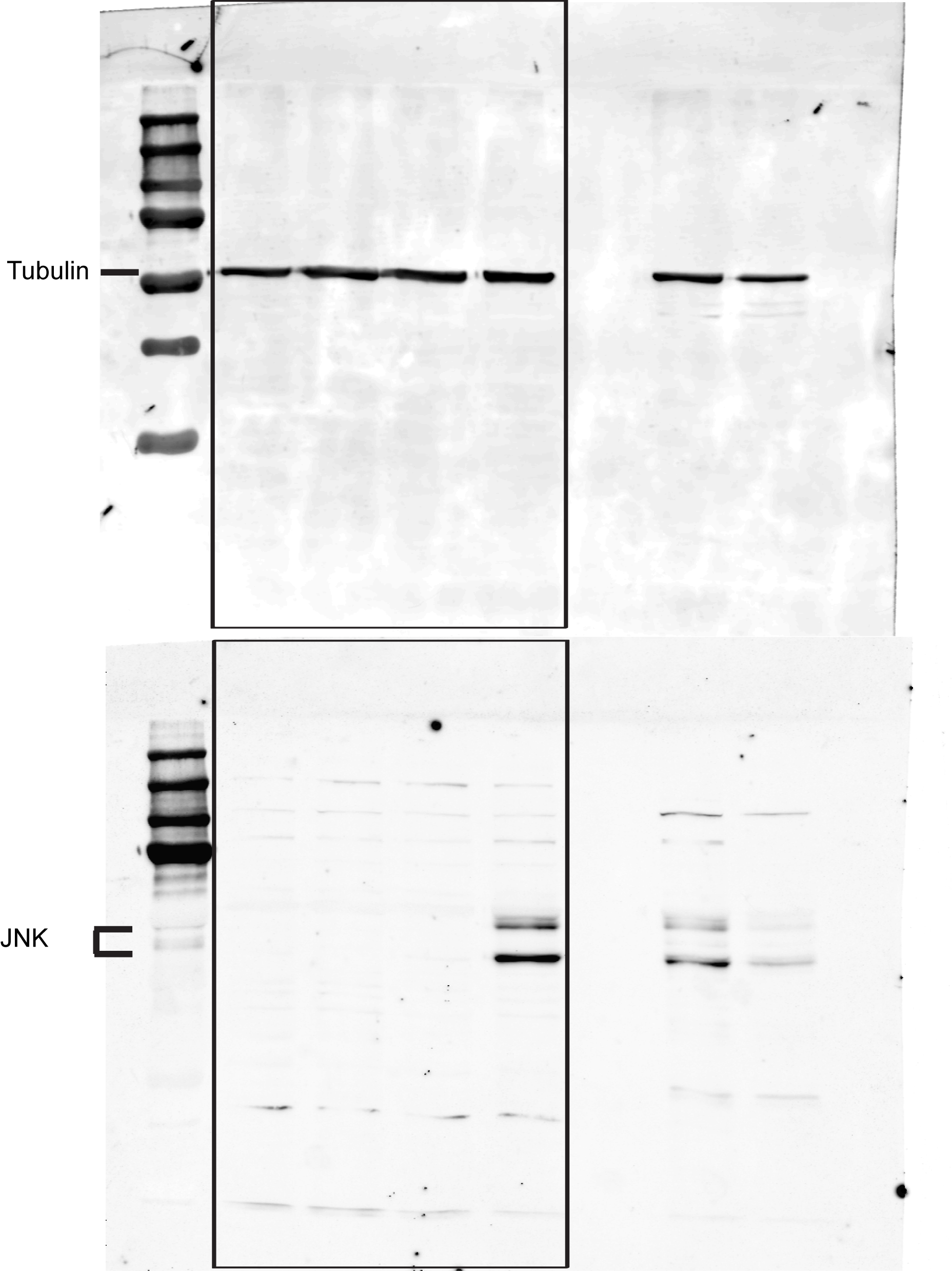

Supplement: Figure 1—source data 2. — Whole immunoblot scans are presented and the region used to construct Figure 1D is outlined. [file elife-36389-fig1-data2.zip › figure1sourcedata2.tif]

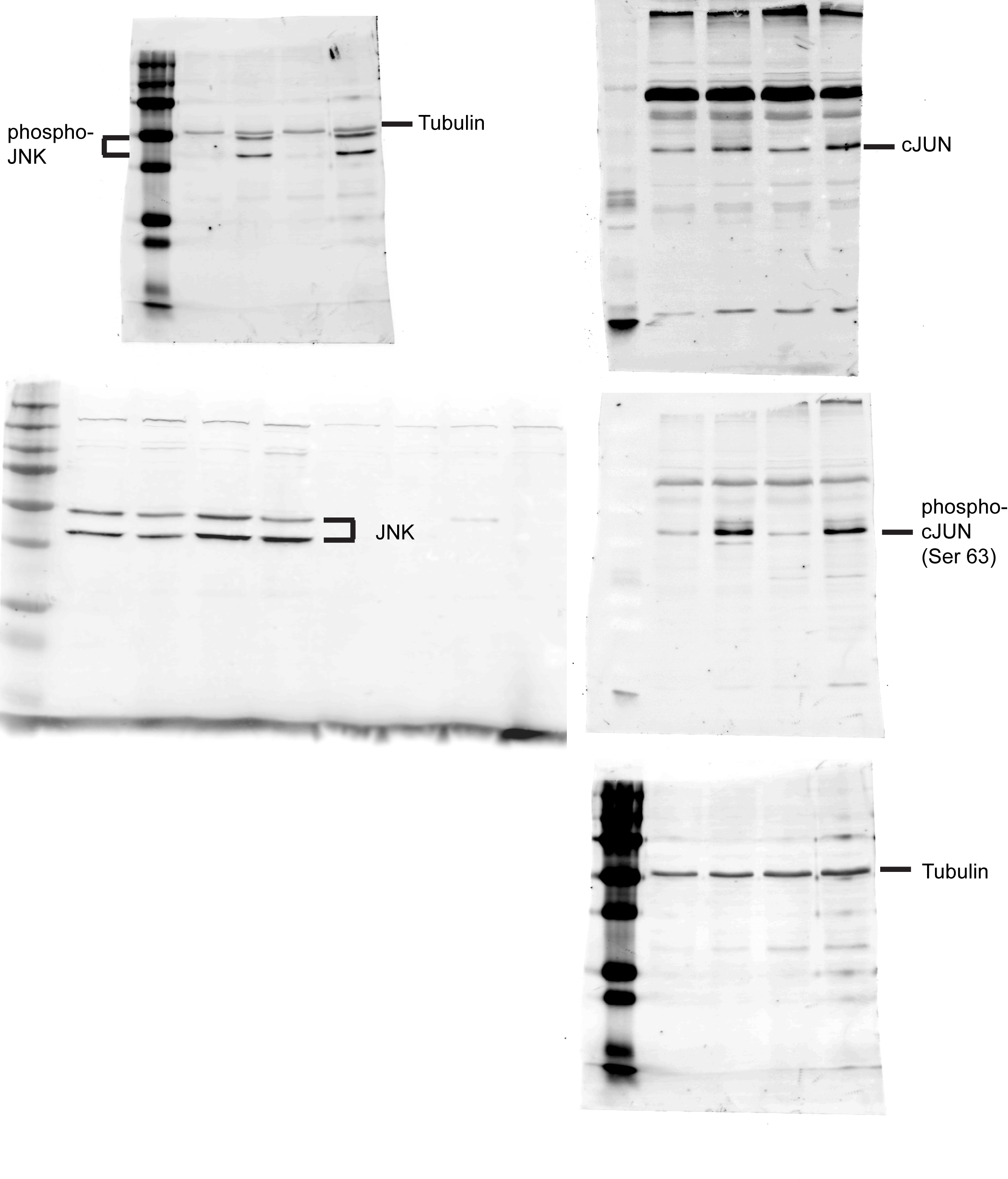

Supplement: Figure 4—figure supplement 2—source data 1. — Whole immunoblot scans are presented and the region used to construct Figure 4—figure supplement 2A is outlined. [file elife-36389-fig4-figsupp2-data1.zip › figure4supp2Asourcedata.tif]

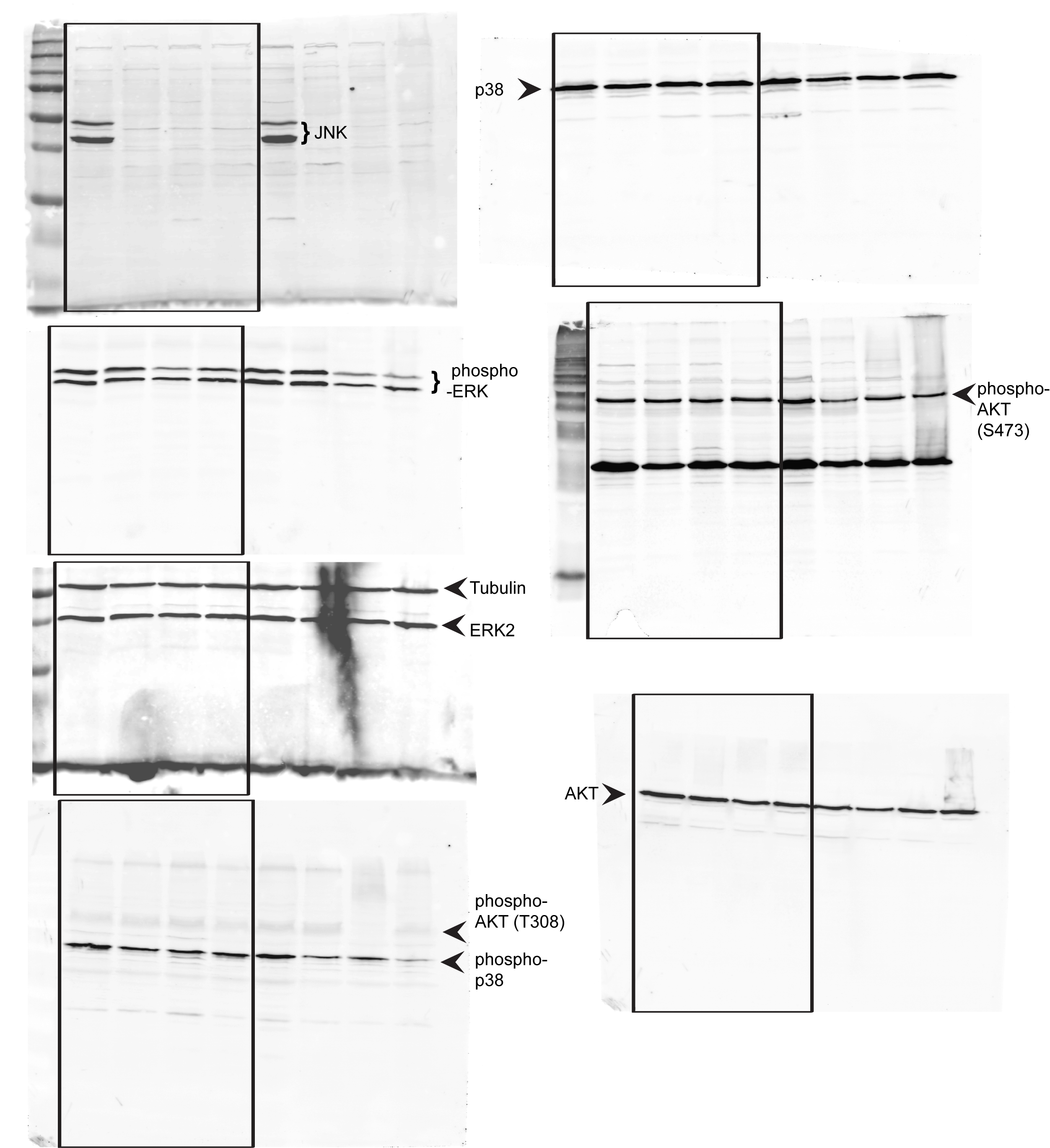

Supplement: Figure 4—figure supplement 2—source data 2. — Whole immunoblot scans are presented and the region used to construct Figure 4—figure supplement 2B is outlined. [file elife-36389-fig4-figsupp2-data2.zip › figure4supp2Bsourcedata.tif]
