# Supplementary material for: The cJUN NH2-terminal kinase (JNK) signaling pathway promotes genome stability and prevents tumor initiation
Source: eLife. 2018 Jun 1;7:e36389. doi: 10.7554/eLife.36389 (PMC5984035; doi:10.7554/eLife.36389)
Supplement: Transparent reporting form [file elife-36389-transrepform.docx]

***eLife’s* transparent reporting form**

We encourage authors to provide detailed information *within their submission* to facilitate the interpretation and replication of experiments. Authors can upload supporting documentation to indicate the use of appropriate reporting guidelines for health-related research (see [EQUATOR Network](http://www.equator-network.org/%20)), life science research (see the [BioSharing Information Resource](https://biosharing.org/)), or the [ARRIVE guidelines](http://www.plosbiology.org/article/info:doi/10.1371/journal.pbio.1000412) for reporting work involving animal research. Where applicable, authors should refer to any relevant reporting standards documents in this form.

If you have any questions, please consult our Journal Policies and/or contact us: [editorial@elifesciences.org](mailto:editorial@elifesciences.org).

**Sample-size estimation**

- You should state whether an appropriate sample size was computed when the study was being designed
- You should state the statistical method of sample size computation and any required assumptions
- If no explicit power analysis was used, you should describe how you decided what sample (replicate) size (number) to use

Please outline where this information can be found within the submission (e.g., sections or figure legends), or explain why this information doesn’t apply to your submission:

Sample sizes for the mouse tumor cohorts were estimated based on power analysis indicating that groups of 17 mice are required to detect differences >1.0 SD (α=0.05). This estimate assumes that the phenotypes of the different groups of mice exhibit equal variance.

RNA-seq studies were performed using 3 biological replicates based on previous experience. Exome sequencing was performed on all of the tumor cell lines that were isolated and genomic DNA isolated from the mammary gland of a virgin mouse of the same genotype. Tissue culture studies were performed using at least two independently isolated cell cultures per group. This information is presented in the Figure Legends.

**Replicates**

- You should report how often each experiment was performed
- You should include a definition of biological versus technical replication
- The data obtained should be provided and sufficient information should be provided to indicate the number of independent biological and/or technical replicates
- If you encountered any outliers, you should describe how these were handled
- Criteria for exclusion/inclusion of data should be clearly stated
- High-throughput sequence data should be uploaded before submission, with a private link for reviewers provided (these are available from both GEO and ArrayExpress)

Please outline where this information can be found within the submission (e.g., sections or figure legends), or explain why this information doesn’t apply to your submission:

The number of biological replicates (e.g. mouse numbers and independently isolated cells), technical replicates, and the number of replication studies are stated in the Figure Legends. Statistical analysis was based on the number of biological replicates. The data associated with each Figure are included in the Source Data file published with this manuscript.

The Kaplan Meier analysis of mammary tumor-free survival was performed by monitoring breast tumor development by palpation. Animals euthanized before a palpable mammary tumor had formed were censored from the analysis. These censored mice are graphically illustrated in Figure 3A.

RNA-seq data have been deposited with NCBI; GEO accession numbers GSE100581 and GSE92560. Exome sequence data have been deposited with NCBI; SRA accession number PRJN401332.

**Statistical reporting**

- Statistical analysis methods should be described and justified
- Raw data should be presented in figures whenever informative to do so (typically when N per group is less than 10)
- For each experiment, you should identify the statistical tests used, exact values of N, definitions of center, methods of multiple test correction, and dispersion and precision measures (e.g., mean, median, SD, SEM, confidence intervals; and, for the major substantive results, a measure of effect size (e.g., Pearson's r, Cohen's d)
- Report exact p-values wherever possible alongside the summary statistics and 95% confidence intervals. These should be reported for all key questions and not only when the p-value is less than 0.05.

Please outline where this information can be found within the submission (e.g., sections or figure legends), or explain why this information doesn’t apply to your submission:

Statistical methods are described in the Methods Section of the manuscript. The specific statistical test, the exact value of n, and the exact value of p, p_adj_, and q (when <0.05) for each experiment are described in the text of the Results Section or Figure Legends of the manuscript. Data associated with each Figure are included in the Source Data file published with this manuscript.

(For large datasets, or papers with a very large number of statistical tests, you may upload a single table file with tests, Ns, etc., with reference to sections in the manuscript.)

**Group allocation**

- Indicate how samples were allocated into experimental groups (in the case of clinical studies, please specify allocation to treatment method); if randomization was used, please also state if restricted randomization was applied
- Indicate if masking was used during group allocation, data collection and/or data analysis

Please outline where this information can be found within the submission (e.g., sections or figure legends), or explain why this information doesn’t apply to your submission:

Groups of mice were assigned based on genotypes. Tissue culture studies were performed by random assignment of cultures to each treatment group. Pathological examination of tissue sections was performed in a blinded manner.

**Additional data files (“source data”)**

- We encourage you to upload relevant additional data files, such as numerical data that are represented as a graph in a figure, or as a summary table
- Where provided, these should be in the most useful format, and they can be uploaded as “Source data” files linked to a main figure or table
- Include model definition files including the full list of parameters used
- Include code used for data analysis (e.g., R, MatLab)
- Avoid stating that data files are “available upon request”

Please indicate the figures or tables for which source data files have been provided:

Flow cytometry data have been deposited with FlowRepository (Repository ID:FR-FCM-ZYEV). RNA-seq data have been deposited with NCBI; GEO accession numbers GSE100581 and GSE92560. Exome sequence data have been deposited with NCBI; SRA accession number PRJN401332. Data associated with each Figure are included in the Source Data file published with this manuscript.
